# Foods for Special Medical Purposes in Home Enteral Nutrition-Clinical Practice Experience. Multicenter Study

**DOI:** 10.3389/fnut.2022.906186

**Published:** 2022-07-07

**Authors:** Marcin Folwarski, Stanisław Kłęk, Agata Zoubek-Wójcik, Waldemar Szafrański, Lidia Bartoszewska, Krzysztof Figuła, Marlena Jakubczyk, Anna Jurczuk, Zbigniew Kamocki, Tomasz Kowalczyk, Bogna Kwella, Przemysław Matras, Joanna Sonsala-Wołczyk, Jacek Szopiński, Krystyna Urbanowicz, Anna Zmarzły

**Affiliations:** ^1^Department of Clinical Nutrition and Dietetics, Medical University of Gdańsk, Gdańsk, Poland; ^2^Home Enteral and Parenteral Nutrition Unit, Department of General Surgery, Nicolaus Copernicus Hospital, Gdańsk, Poland; ^3^Surgical Oncology Clinic, Maria Skłodowska-Curie National Cancer Institute, Kraków, Poland; ^4^Nutrimed Home Nutrition Center, Rzeszow, Poland; ^5^First Department General and Transplant Surgery and Clinical Nutrition Medical University of Lublin, Home Enteral and Parental Nutrition Unit SPSK4, Lublin, Poland; ^6^Nutricare Clinical Nutrition Center, Kraków, Poland; ^7^Department of Anaesthesiology and Intensive Care Collegium Medicum in Bydgoszcz, Nicolaus Copernicus University in Toruń, Toruń, Poland; ^8^Nutritional Team, Home Enteral and Parenteral Nutrition Clinic University Hospital No. 1 in Bydgoszcz, Toruń, Poland; ^9^Outpatient Clinic of Nutritional Therapy Clinical Hospital of Białystok, Białystok, Poland; ^10^2nd Department of General, Gastroenterological and Oncological Surgery Medical University of Białystok, Białystok, Poland; ^11^Department of Clinical Nutrition, Provincial Specialist Hospital, Olsztyn, Poland; ^12^Gromkowski City Hospital Wrocław, Clinical Nutrition Unit, Wrocław, Poland; ^13^Department of General Hepatobiliary and Transplant Surgery, Collegium Medicum in Bydgoszcz, Nicolaus Copernicus University in Toruń, Toruń, Poland; ^14^General Surgery and Clinical Nutrition Ward, Community Hospital Bydgoszcz, Nicolaus Copernicus University in Toruń, Toruń, Poland

**Keywords:** home enteral nutrition (HEN), enteral nutrition, enteral formulas, FSMP, protein provision, micronutrients, vitamin, energy

## Abstract

**Background:**

Enteral nutrition (EN) with foods for special medical purposes (FSMP) is recommended for most patients on home enteral nutrition (HEN). Although there are disease-specific guidelines for energy, protein, and micronutrient provision, only a few studies are showing real-life experience in the long-term use of FSMP.

**Methods:**

In a multicenter study, the influence of the FSMP composition and administration technique (bolus vs. continuous) on protein and energy provision in HEN was analyzed. Provision of vitamins and minerals was compared to recommended daily allowance (RDA) and upper tolerable limit (UL).

**Results:**

Approximately, 772 patients on HEN, mostly (88.6%) with oncological and neurological diseases, were enrolled. The patients on standard FSMP received less protein and energy than those on hypercaloric and protein enriched despite receiving higher volumes of EN (*p* < *0.05*). No differences were observed in jejunal feeding with oligomeric vs. polymeric FSMP in terms of energy, protein, and volume. Continuous gastric feeding provided more protein, energy, and volume vs. bolus feeding (*p* < *0.05*). Significant number of patients received less than 100% RDA of vitamin D (50.5%), vitamin B3 (49%), vitamin K (21.8%), vitamin B5 (64.3%), vitamin B9 (60%). Majority of the patients received less than 100% RDA of sodium (80.2%), potassium (99%), chloride (98%), calcium (67%), magnesium (87%), fluoride (99%), and iodine (43%). Approximately, 43.63% of cancer and 49.9% of neurological patients received less than 1 g/kg/day of protein and 51.7% of cancer and 55.5% of neurological patients received less than 25 kcal/kg/day.

**Conclusion:**

Awareness of the available compositions of FSMP and advantageous profiles of specific diets may lead to the implementation of recommendations for EN. HEN professionals need to analyze all the patient’s needs and requirements to provide more tailored matching of nutritional support.

## Introduction

The prevalence of home enteral nutrition (HEN) is growing, which was demonstrated in several studies from Europe and the United States (1–5). The most common indications for HEN are oncological and neurological diseases (6). The rates of patients with cancer, especially with head and neck cancer (HNC) and upper gastrointestinal (GI) tract cancer, are growing (7, 8). Malnutrition affects both neurological and oncological patients, and proper nutritional support is crucial to improve the comprehensive treatment.

Recommended protein intake for patients with cancer according to the European Society for Clinical Nutrition and Metabolism (ESPEN) is at least 1 g/kg/day (1.5 g/kg/day if possible) and energy provision between 25 and 30 kcal/kg/day (9). Vitamins and minerals should be provided in recommended daily doses for the general population with no indications for additional supplementation in patients with no proven deficiencies. Most neurological patients require similar energy provision with disease-specific recommendations for protein supply (mostly 1 g/kg/day). In some neurological diseases, such as Parkinson’s disease or multiple sclerosis, monitoring of Vitamin D, folic acid, and Vitamin B12 is recommended because some micronutrient deficiencies may contribute to the onset or progression of the diseases (10, 11). Studies have shown that commercially available formulas/foods for special medical purposes (FSMP) make it possible to meet recommendations and facilitate planning of nutritional support. Therefore, they are recommended by ESPEN in HEN (12, 13) unless there is a specific rationale for mixed diets. A Polish study showed that FSMP and the support of nutrition teams (NST) are cost-effective and improve clinical outcomes (14). The composition of FSMP is regulated by legal documents of the European Union (EU). EU Commission regulates the microelement content of FSMP in a directive 1999/21/EC (15), and European Food Safety Authority (EFSA) sets upper tolerable doses of nutrients (UL) (16, 17).

Studies show that EN intolerance and complications manifested with gastrointestinal symptoms like diarrhea, nausea, delayed gastric emptying, abdominal pain, constipation, or bloating may lead to underdosing of FSMP and underfeeding of the patients (18). Those observations come from data on hospitalized patients; however, little is known on EN in long-term or home conditions. In Poland, HEN is reimbursed only for patients on FSMP, which enables homogenous scientific considerations on the use of commercially available formulas. Our study aimed to confront the real-life clinical experience of using FSMP in HEN with recommendations and guidelines for clinical nutrition concerning protein, energy, and micronutrient provision.

## Materials and Methods

This multicenter study was conducted in 22 Polish HEN centers. Data on adult patients on HEN treated between January 1 and December 31, 2018 were retrospectively evaluated. We included all tube-fed patients on FSMP for at least 7 days (with no or negligible oral intake) and complete medical history containing basic anthropometric data (weight, height), main indications for HEN, route for nutritional support, provision technique, and administered FSMP. Types of FSMP, total volume, protein, energy, and micronutrient composition [vitamin A, vitamin D, vitamin E, vitamin K, vitamin B1 (thiamin), vitamin B2 (riboflavin), vitamin B3 (niacin), vitamin B6, vitamin B9 (folate), vitamin B12, vitamin B7 (biotin), vitamin C, sodium, potassium, chloride, calcium, phosphorus, magnesium, iron, zinc, copper, manganese, fluoride, molybdenum, selenium, chromium, iodine] were collected from medical records. Study coordinators of the participating HEN centers were asked to provide the most recent data during the observation period. We excluded patients treated with supplemental parenteral nutrition. Data were anonymized and the study protocol was approved by the Local Ethics Committee (KB-7/20).

The aim of the study was to assess the influence of the FSMP type and the method of administration (bolus vs. continuous) on protein and energy provision. Administration of vitamins and minerals with FSMP was analyzed. FSMP were classified according to the following definitions: hypercaloric (HC) ≥ 1.3 kcal/ml, protein-enriched (PE) > 4 g/100 ml, standard (STD)- polymeric, and isocaloric. Bolus vs. continuous provision was compared in patients with gastric access. Oligomeric vs. polymeric FSMP in patients with feeding jejunostomies. Gender-specific micronutrient recommended daily allowance (RDA) and tolerable upper intake level (UL) were established according to EU regulations (15–17) and nutritional standards for the Polish population (19) (Supplementary Table 4 in Supplementary Material).

### Statistical Analysis

The calculations were carried out with the use of the Statistica 13 package and Microsoft Excel 2013. The descriptive statistics include averages, medians, and standard deviations (SD). The quantitative variables were characterized by the arithmetic mean of standard deviation or median or max/min (range) and 95% confidence interval. The qualitative variables were presented with the use of count and percentage. To check if a quantitative variable derives from a population of normal distribution, the Shapiro–Wilk test has been used. Whereas to prove the hypotheses on the homogeneity of variances, Leven (Brown–Forsythe) test has been utilized. Statistical significance of differences between the two groups (the un-paired variables model) was processed with the Student’s T-test (or Welch test in the case of a lack of homogeneity) or U Mann–Whitney test (in cases where conditions of performing the Student’s *T*-test were not satisfied or for variables measured by ordinal scale). The significance of the differences between more than two groups was assessed with F-test (ANOVA) or Kruskal–Wallis (if AVOVA conditions were not fulfilled). In the case of statistically significant differences between the two groups, *post hoc* tests were utilized (Tukey test for F or Dunn for Kruskal–Wallis). Chi-squared tests for independence were used for qualitative variables (with the use of Yates correction for cell counts below 10, with a check of Cochrane’s conditions or with Fisher’s exact test, respectively). To determine dependence, strength, and direction between variables, correlation analysis was used by determining the Pearson or Spearman’s correlation coefficients. In all the calculations, the statistical significance level of *p* = *0.05* has been used.

## Results

Approximately, 772 patients (45.21%, female; 54.79%, male) with median age of 63.65 years (SD ± 21.9); weight, 55 kg (SD ± 19.3 kg); and BMI, 20.08 kg/m2 (SD ± 4.66) were enrolled in the study. Neurological or oncological diseases were the primary causes of HEN in 88.6% of patients. Approximately, 90.54% of patients were treated with gastric access (98.12% of neurological, 77.99% of cancer, 90.91% of other) (Table 1). Patient groups with the highest protein and energy supply were cystic fibrosis, muscle dystrophies, cerebral palsy, and multiple sclerosis. The lowest protein and energy provision were encephalopathies, neurovascular diseases (mainly stroke), GI cancer, and amyotrophic lateral sclerosis (Supplementary Table 1 in Supplementary Material).

**TABLE 1:** Feeding access.

	Other (*n* = 88)	Neurological (*n* = 425)	Cancer (*n* = 259)	All (*n* = 772)
				
	(n;%)	(n;%)	(n;%)	(n;%)
PEG	66; 75	337; 79.29	158; 61	561; 72.67
Gastrostomy	3; 3.14	13; 3.06	31; 11.97	47; 6.09
Naso-gastric tube	11; 12.5	67; 15.76	13; 5.02	91; 11.79
PEG-PEJ	1; 1.14	5; 1.18	2; 0.77	8; 1.04
Naso-jejunal tube	3; 3.41	0; 0	3; 1.16	6; 0.78
Jejunostomy	4; 4.55	3; 0.71	52; 20.08	59; 7.64

*PEG- percutaneous endoscopic gastrostomy.*

*PEG-PEJ- percutaneous endoscopic transgastric jejunostomy.*

### Volume, Protein, and Energy Supply

**FIGURE 1:**
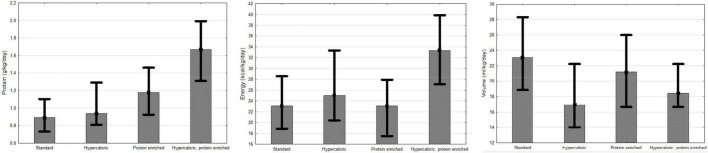
Volume, protein, and energy provision of FSMP.

The patients were administered a median total calory provision of 1,250 kcal/day (SD ± 364 kcal); protein, 55.9 g/day (SD ± 19.3 g/day); and volume of 1,000 ml/day (SD ± 268 ml). Median protein provision in patients on STD (0.89/kg/day) was not statistically different from HC (0.94/kg/day) (*p* = 0.062). PE provided more protein (1.18/kg/day) than STD (p < 0.001) and HC (*p* = 0.0189). Hypercaloric and protein-enriched formulas (HC-PE) provided more protein (1.67/kg/day) than HC (*p* < 0.001), PE (*p* < 0.001), and STD (*p* < 0.001). The patients on STD were administered higher volumes of EN (23.08 ml/kg/day) than on HC (16.95 ml/kg/day) (*p* < 0.001), PE (21.18 ml/kg/day) (*p* = 0.0089), and HC-PE (18.45 ml/kg/day) (*p* < 0.001). Energy provision for the patients on HC and PE (33.33 kcal/kg/day) was higher than in STD (23.08 kcal/kg/day), HC (25 kcal/kg/day) and PE (23.08 kcal/kg/day) (Figure 1). Similar differences were observed in the subgroup of the patients with cancer; however, the patients on STD were administered similar volume of EN to PE (23.08 vs. 23.81, *p* = 1.). The neurological patients on PE received less energy than STD, HC, and HC-PE (20 vs. 23.08, 25, 33.33 kcal/kg/day). Approximately, 45.9% of all the patients were provided with less than 1 g/kg/day and 52.46% received less than 25 kcal/kg/day (Table 2 and Supplementary Figures 1-3 in Supplementary Material).

**TABLE 2:** Protein and energy provision.

	Other (*n* = 88)	Neurological (*n* = 425)	Cancer (*n* = 259)	All (*n* = 772)
				
Protein (g/kg)	(n;%)	(n;%)	(n;%)	(n;%)
<1	30; 34.09	212; 49.88	113; 43.63	355; 45.98
1–1,5	40; 45.45	138; 32.47	105; 40.54	283; 36.66
> = 1,5	18; 20.45	75; 17.65	41; 15.83	134; 17.36
**Energy (kcal/kg)**				
<25	35; 39.77	236; 55.53	134; 51.74	405; 52.46
25-30	19; 21.59	79; 18.59	61; 23.55	159; 20.6
> = 30	34; 38.64	110; 25.88	64; 24.71	208; 26.94

**FIGURE 2:**
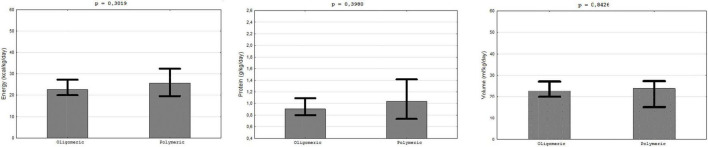
Provision with protein, energy, and volume. Oligomeric vs. polymeric FSMP.

In the patients with jejunal access (*n* = 73), mainly jejunostomy (80.82%) oligomeric (61.64%) and polymeric (38.36%) FSMP were used. No significant differences were observed between oligomeric and polymeric diets in protein (0.91 vs. 1.04 g/kg/day), energy (22.67 vs. 25.57 kcal/kg/day) and total volume of FSMP supply (22.67 vs. 23.82 ml/kg/day) (Figure 2).

### Administration Method

**FIGURE 3:**
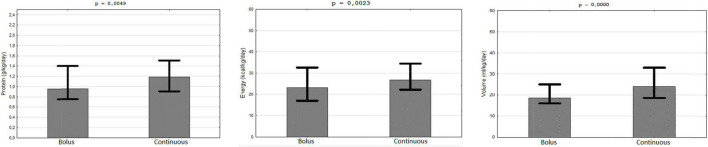
Provision of protein, energy, and volume of FSMP. Bolus vs. continuous feeding.

The patients with gastric access (*n* = 364) used bolus (74.6%) and continuous (24.45%) feeding. Approximately, 50% of the patients on continuous used pumps. Continuous feeding provided more protein (1.19 vs.96 g/kg/day), energy (26.67 vs. 23.08 kcal/kg/day), and volume (24 vs. 18.57 ml/kg/day) in FSMP (Figure 3).

**FIGURE 4:**
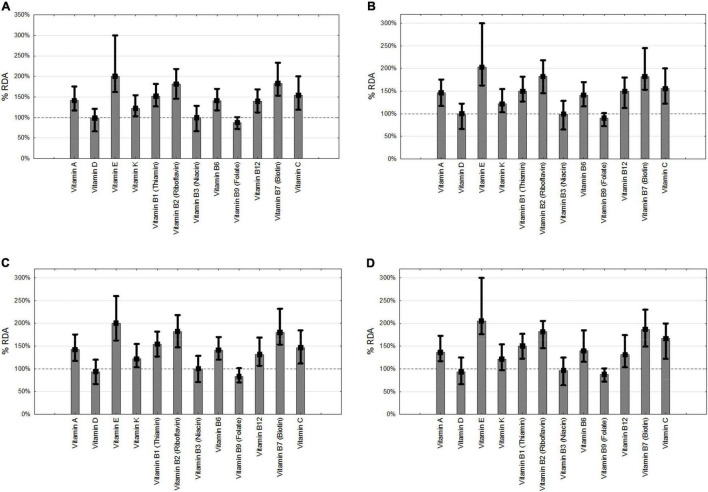
Provision of vitamins. **(A)** all patients, **(B)** neurology, **(C)** cancer, **(D)** others. RDA- recommended daily allowance.

**FIGURE 5:**
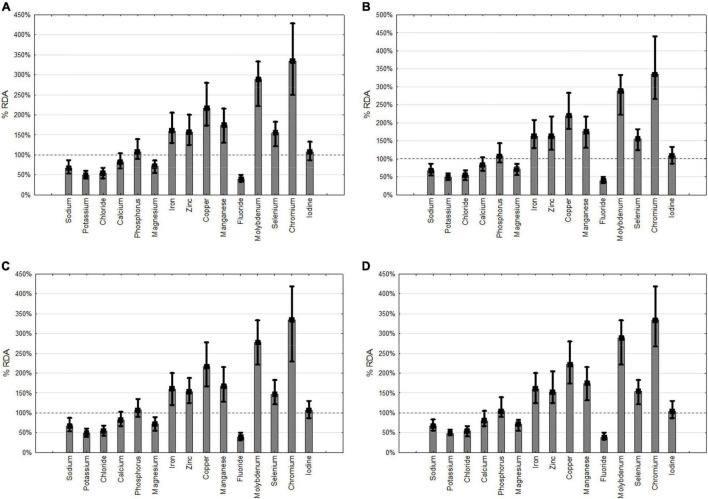
Provision of minerals. **(A)** all patients, **(B)** neurology, **(C)** cancer, **(D)** others.RDA- recommended daily allowance.

The majority of the patients with HEN received more than 100% of RDA of vitamins A, E, K, B1, B12, C and phosphorus, iron, zinc, copper, manganese, molybdenum, selenium, and chromium. Tolerable upper intake levels (UL) were exceeded in.13% of the patients in vitamin A, 4.27% vitamin D, 0.13% vitamin B9, 1.04% for calcium, 55.7% magnesium, 6.35% zinc, 0.13% copper. No patients were treated with doses exceeding UL with vitamin E, B6, molybdenum, selenium, and iodine. No ULs were provided by EFSA for vitamin K, B1, B2, B3, B5, B12, B7, C, sodium, potassium, chloride, phosphorus, iron, manganese, fluorine, and chromium (Figures 4, 5).

## Discussion

HEN in Poland is supervised by specialistic NST, consisting of physicians, nurses, and in units based on the hospital background, also dietitians and pharmacists. Nevertheless, recommended calorie and protein provisions were not reached in a significant number of the patients in our study. More than 43% of the cancer and nearly half of the neurological patients received less than 1g/kg/day of protein and more than half of the cancer and neurological patients received less than 25 kcal/kg/day. It is said that most patients receiving long-term nutritional support benefit from standard diets. However, we have observed that they receive less protein and less energy than those receiving hypercaloric formulas, despite higher amounts of EN compared with those receiving protein enriched. A Spanish study by Ballesteros Pomar and colleagues showed that long-term provision of hypercaloric and protein-enriched diet is associated with improving tolerance of EN and quality of life of cancer and neurological patients (20). In the ICU setting, hypercaloric diets, although enable higher energy provision, are associated with more common regurgitation, vomiting, and higher gastric residual volume with no differences in protein provision and 90-day mortality (21). Interestingly, energy intake in our study was highest in adult patients with cerebral palsy (35 kcal/kg/day). Although data show that the resting metabolic rate of patients with cerebral palsy (CP) may be higher than that of healthy controls (22), most researchers admit that total energy requirements may even be lower than average in most cases because of lower activity due to disability (22–26). Future studies are needed to assess whether long-term nutritional support with high energy intake leads to positive clinical outcomes CP.

Although there are no scientific proofs or guidelines to use oligomeric formulas for EN administered through jejunal access, many practitioners prefer this strategy. We found no difference in protein, energy, or total volume provision between patients receiving polymeric and oligomeric FSMP. However, we are aware that this was an observational study, and the reasons for FSMP choice were not analyzed. The patients on oligomeric diets could have had a previous history of EN intolerance and consequent FSMP modifications. Nevertheless, those observations show that polymeric formulas can be used in patients with jejunal feeding with a similar provision. The influence of oligomeric diets on EN tolerance is not clear, with some studies showing GI symptoms reduction (27, 28) and others showing no influence (29, 30). In studies on experimental models, the absorption of energy and nutrients did not differ between oligo and polymeric diets. Polymeric diets though they had lower osmolarity induced similar intestinal water secretion due to rapid pancreatic hydrolysis. The authors underlined that the osmolarity of diet plays an important role in EN, however, mainly in enteric feeding (31). Several physiological mechanisms in the GI tract are regulating the passage and digestion of the diet and fluids. Energy density, volume, osmolarity, pH, and other factors influence gastric emptying; consequently, the composition of FSMP may not be as valid for patients on gastric feeding, especially in the topic of diarrhea prevention (32). In a recently published consensus survey, surgeons agreed that oligomeric formulas may be useful for surgical patients with short bowel syndrome in the postoperative phase, for patients with diarrhea or malabsorption, after upper gastrointestinal surgery or when the feeding tube is placed distally to the duodenum (33). Other studies suggest the use of oligomeric oral and enteral FSMP for the prevention and treatment of oncology treatment-related diarrhea (34). Most studies on oligomeric diets were conducted in the hospital setting mainly on ICU patients consequently with short-term observations. Study on community EN showed that new high-energy, high-protein and peptide-based formulas can lead to good tolerability and higher energy and protein provision (35). The transition from polymeric formulas to peptide-based formulas reduced gastrointestinal symptoms and health care utilization in HEN patients with EN intolerance in the Mayo Clinic study (36).

Continuous feeding is recommended for EN in patients with jejunal accesses. Patients with gastric access on the other hand can be treated with a bolus or continuous feeding. In our data continuous feeding was associated with a higher protein, energy, and total volume provision in patients with gastric EN. To our best knowledge, there are no prospective randomized studies in long-term HEN to confirm those results. Continuous feeding didnŠt influence the small bowel water fluid content in healthy volunteers; therefore, it is not clear whether this factor may contribute to EN-related diarrhea (37). No difference in tolerance and outcomes was observed in acute stroke patients on EN with continuous vs intermitted feeding (38). On the other hand, the adjusted infusion rate of EN with a feeding pump reduced the risk of respiratory complications caused by aspiration in post-stroke patients requiring EN (39). There was no difference in calorie intake between continuous and bolus feeding in critical illness patients (40). A recent meta-analysis on ICU patients showed that continuous feeding was associated with decreased feeding intolerance and incidence of high gastric residual volume and aspiration risk. However, intermitted feeding was associated with reduced constipation and a higher amount of calory provision (41). Bolus feeding is considered a more physiological method of gastric feeding, easier to manage for physically active patients. Heyland studies show that enteral nutrition intolerance may affect 24% and reduce the intake by 10%. However, those patients were treated in ICU, and intolerance was mostly observed during the first days on EN (18).

Since the median provision of FSMP in our study was relatively low (1,000 ml, and 1,250 kcal/day) underdosing of micronutrients was also expected. Nevertheless, we found that administered vitamin levels were mostly above 100% of RDA. On the other hand, nearly half of the patients did not reach recommended doses of vitamin D, B3, B5, and B9, and more than 20% were not given RDA of vitamin K. Majority of patients received less than RDA of sodium, chloride, calcium, and fluoride. However, those data need to be analyzed with caution since additional fluid administration was not included in the analysis. Tolerable Upper Intake Levels were exceeded in only a few patients considering vitamins A and D. We observed that molybdenum, chromium, copper, and manganese were administered significantly above RDA. EFSA does not provide UL for vitamin K, B1, B2, B3, B5, B12, B7, C, sodium, potassium, chloride, phosphorus, iron, manganese, fluoride, and chromium. In the study of Iacone and colleagues, more than sixty enteral formulas were compared for micronutrients. In some formulas, fluorine and vitamin K requirements were not reached, and on the other hand, other micronutrients like vitamin A, zinc, manganese, and chromium were overdosed in patients receiving 1,500 and 2,000 kcal/day (42). In the study on the Italian HEN population, the macronutrient and micronutrient requirements of the local society of nutrition were covered except for potassium, fluoride, and vitamin K. Mean protein provision was 67 and 63g/day for males and females, respectively. Overdosing of Fe, Zn, Cu, Se, vitamin A, E, B1, B2, B6, and B12 was observed (43). In the study of Henderson, 58% of patients received RDA of vitamins and minerals in enteral nutrition despite median calorie intake being 37.7 kcal/kg/day and 1.4 kg/kg/day of protein. In this study at the time of qualification, 65% received multivitamin supplementation (44).

### Study Limitations and Advantages

There are only a few studies showing the real-life experience of FSMP with the discussion on macro and micronutrient provision. Multicenter data collection provides more universal conclusions not influenced by local strategies of NSTs in HEN units. However, due to the observational and retrospective design of the study, it has some limitations. Although we observed interesting conclusions on oligomeric vs. polymeric FSMP or continuous vs. bolus feeding, this was not a randomized prospective study; consequently, hypothetical conclusions rather than definitive statements can be drawn. We additionally did not collect data on FSMP intolerance or any other possible reasons for the selection of FSMP. Moreover, we collected data from medical records, and we were not able to obtain confirmation from the patients about actual diet intakes. Although we analyzed micronutrient provision, we did not collect data on additional supplementation and can only discuss the FSMP-derived sources for vitamins and minerals. No information on the specific health status of the patients was included; consequently, we based on general recommendations for the cancer and neurological patients. We are aware that for patients with cancer, in palliative treatment, the goals and requirements, as well as tolerance of HEN, are different. Similarly, neurological patients may require less energy in their diet due to limited physical activity.

## Conclusion

Protein and energy provision may be insufficient when STDs are used, even though the volume of STDs is higher than in HC and PE. More than half of the patents are provided with less than 1 g/kg/day of protein in STD and HC. Additionally, more than half of patients with cancer are provided less than 1 g/kg/day and 25 kcal/kg/day with STD. It is possible to administer both polymeric and oligomeric diets to jejunostomy, achieving similar protein, energy, and volume administration of EN. Continuous feeding may be beneficial for some patients with gastric feeding and provide more calories, protein, and volume of FSMP. Although FSMP are mostly providing all required macro and micronutrients, more caution is needed to evaluate specific needs for the patient. Knowledge of specific ingredients of FSMP and advantageous profiles of specific diets may lead to meeting the recommendations. HEN professionals need to analyze all patients’ requirements and provide more tailored matching of EN. This study may be a red flag, signaling a need to focus on several clinical factors like monitoring of actual compliance of FSMP and energy and protein provision in HEN.

## Data Availability Statement

The data are available from the corresponding author upon reasonable request.

## Ethics Statement

The studies involving human participants were reviewed and approved by Local Ethics Committee (KB-7/20). Written informed consent for participation was not required for this study in accordance with the national legislation and the institutional requirements.

## Author Contributions

MF contributed to the conceptualization, design of the study, data curation, formal analysis, investigation, statistical and graphical analysis, methodology, resources, supervision, writing—original draft and review and editing. SK contributed to the investigation, methodology, and writing—review and editing. MF, SK, AZ-W, WS, LB, KF, MJ, AJ, ZK, TK, BK, PM, JS-W, JS, KU, and AZ contributed to collection of data. All authors have read and agreed to the published version of the manuscript.

## Conflict of Interest

MF received speaker’s honoraria from: Nutricia, Fresenius Kabi, B Braun, Baxter, Nestle. SK received speaker’s honoraria from: Baxter, B Braun, Fresenius Kabi, Nestle, Nutricia, Takeda, Olimp-Labs. PM received speaker’s honoraria from: Baxter, B Braun, Fresenius Kabi, Nestle, Nutricia, Takeda. WS received speaker’s honoraria from: Nutricia, Fresenius Kabi, B Braun, Baxter, Nestle. AZ-W employed in Nutricia Polska Sp.z o.o. The remaining authors declare that the research was conducted in the absence of any commercial or financial relationships that could be construed as a potential conflict of interest.

## Publisher’s Note

All claims expressed in this article are solely those of the authors and do not necessarily represent those of their affiliated organizations, or those of the publisher, the editors and the reviewers. Any product that may be evaluated in this article, or claim that may be made by its manufacturer, is not guaranteed or endorsed by the publisher.
