# Supplementary material for: Foods for Special Medical Purposes in Home Enteral Nutrition-Clinical Practice Experience. Multicenter Study
Source: Front Nutr. 2022 Jul 7;9:906186. doi: 10.3389/fnut.2022.906186 (PMC9301075; doi:10.3389/fnut.2022.906186)
Supplement: Supplementary file 1 [file Data_Sheet_1.docx]

Supplementary Material

Table 1. FSMP provision in analysed diseases

|  | **Energy (kcal/kg/day)** | | | | | **Protein (g/kg/day)** | | | | **Volume (ml/kg/day)** | | | |
| --- | --- | --- | --- | --- | --- | --- | --- | --- | --- | --- | --- | --- | --- |
| **Primary disease** | **n** | **median** | **Q25** | **Q75** | **SD** | **median** | **Q25** | **Q75** | **SD** | **median** | **Q25** | **Q75** | **SD** |
| **Cancer** | 259 | 24,39 | 20,00 | 29,79 | 7,26 | 1,00 | 0,80 | 1,26 | 0,39 | 21,74 | 17,65 | 26,09 | 6,05 |
| Head and neck | 143 | 25,45 | 21,28 | 31,91 | 7,56 | 1,07 | 0,85 | 1,40 | 0,41 | 22,22 | 17,54 | 26,32 | 6,24 |
| GI | 106 | 22,72 | 18,87 | 27,45 | 6,67 | 0,92 | 0,74 | 1,13 | 0,34 | 20,00 | 17,50 | 25,42 | 5,90 |
| Other (cancer) | 10 | 23,94 | 22,22 | 26,32 | 4,71 | 1,10 | 0,89 | 1,21 | 0,23 | 22,51 | 20,20 | 26,32 | 5,08 |
| **Neurology** | 425 | 23,08 | 18,18 | 30,00 | 15,02 | 0,95 | 0,78 | 1,28 | 0,64 | 21,82 | 16,67 | 27,91 | 12,70 |
| Neurovascular | 110 | 22,68 | 20,00 | 25,00 | 5,49 | 0,90 | 0,80 | 1,09 | 0,26 | 21,68 | 17,86 | 25,00 | 6,21 |
| Other Encephalopathy | 122 | 16,67 | 14,71 | 25,00 | 12,30 | 0,89 | 0,67 | 1,09 | 0,61 | 16,67 | 14,29 | 21,33 | 9,45 |
| Cerebral palsy | 82 | 35,71 | 29,41 | 47,62 | 22,54 | 1,32 | 1,01 | 1,88 | 0,99 | 33,33 | 27,78 | 44,44 | 18,08 |
| Multiple sclerosis | 14 | 26,97 | 20,59 | 30,00 | 5,00 | 1,16 | 0,78 | 1,49 | 0,33 | 26,33 | 20,59 | 29,76 | 4,75 |
| Muscle dystrophy | 9 | 33,33 | 27,03 | 33,33 | 7,32 | 1,33 | 1,08 | 1,50 | 0,37 | 27,78 | 24,76 | 33,33 | 7,92 |
| Neurodegenerative | 33 | 23,08 | 20,00 | 26,67 | 5,43 | 0,96 | 0,81 | 1,18 | 0,30 | 23,08 | 20,00 | 26,67 | 5,39 |
| Amyotrophic Lateral Sclerosis | 43 | 21,82 | 18,57 | 26,00 | 9,79 | 0,93 | 0,73 | 1,33 | 0,53 | 20,83 | 17,86 | 24,00 | 6,87 |
| Other neurological | 21 | 23,44 | 18,33 | 30,74 | 11,34 | 0,91 | 0,76 | 1,19 | 0,52 | 21,43 | 18,07 | 25,00 | 9,81 |
| **Other** | 88 | 26,01 | 21,90 | 32,52 | 7,76 | 1,05 | 0,84 | 1,35 | 0,39 | 23,26 | 19,23 | 27,89 | 6,59 |
| Cystic fibrosis | 11 | 29,41 | 22,22 | 36,59 | 9,44 | 1,43 | 0,96 | 1,70 | 0,50 | 20,00 | 17,48 | 24,39 | 7,39 |
| Trauma | 18 | 25,00 | 22,06 | 30,77 | 5,82 | 1,02 | 0,93 | 1,26 | 0,28 | 23,08 | 17,50 | 25,00 | 4,00 |
| Non-cancer gastric and esophageal dysfunctions | 22 | 25,83 | 21,74 | 30,30 | 7,64 | 1,01 | 0,90 | 1,24 | 0,38 | 22,23 | 20,55 | 28,00 | 5,96 |
| Other | 28 | 25,00 | 19,74 | 32,09 | 8,30 | 0,95 | 0,77 | 1,28 | 0,39 | 25,00 | 19,44 | 29,56 | 7,07 |


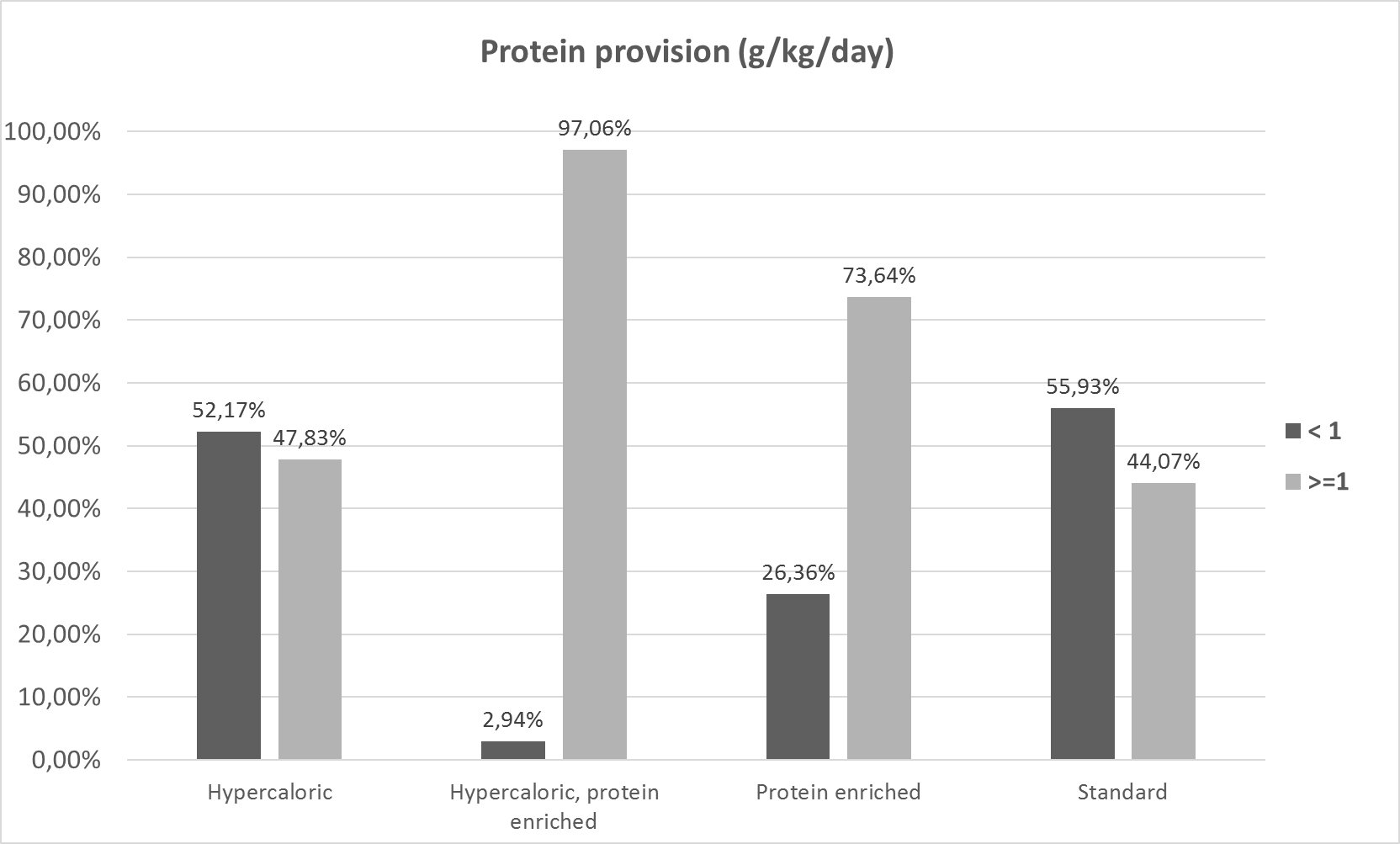


Figure 1. Protein provision and FSMP type in general population


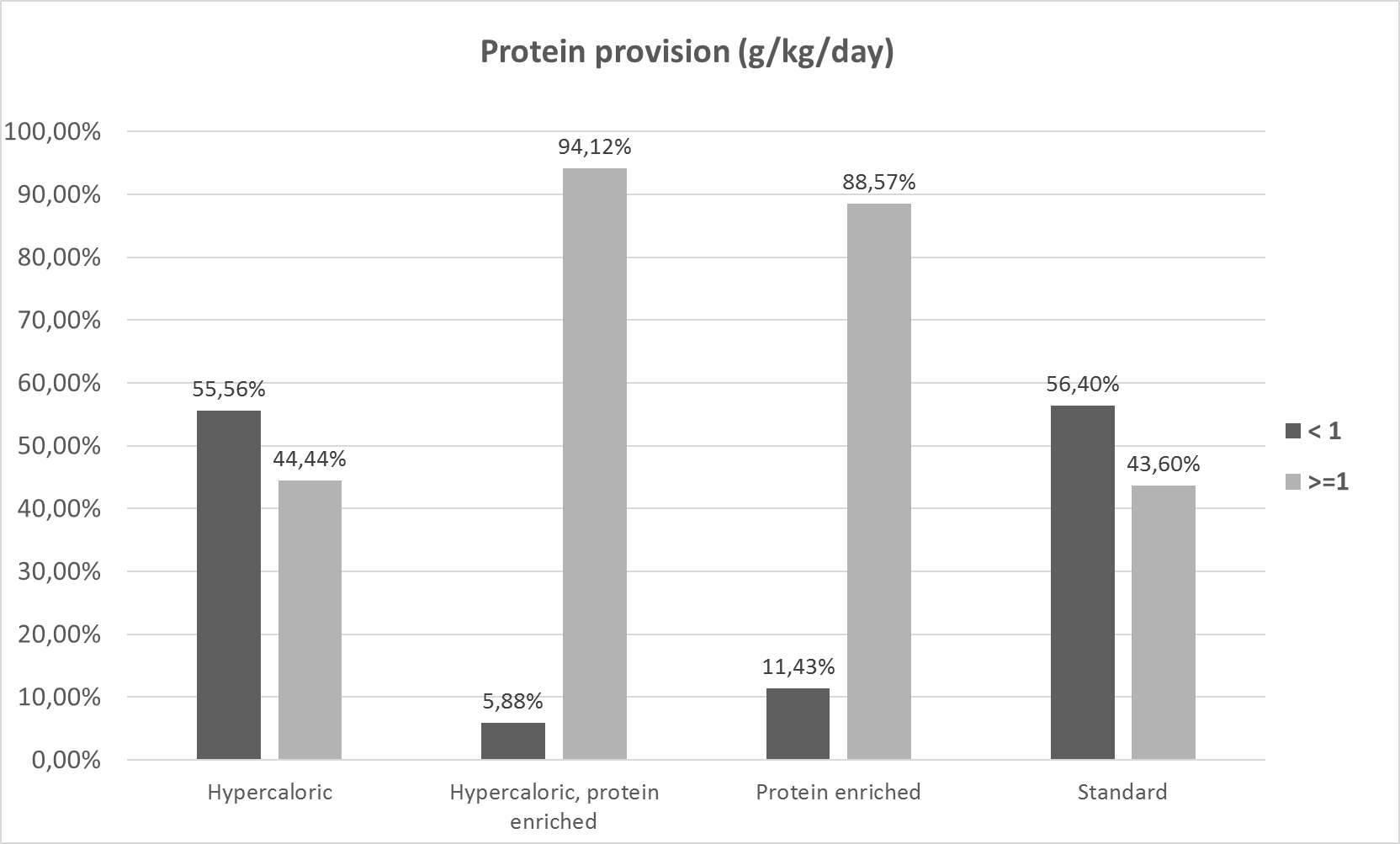


Figure 2. Protein provision and FSMP type in cancer patients


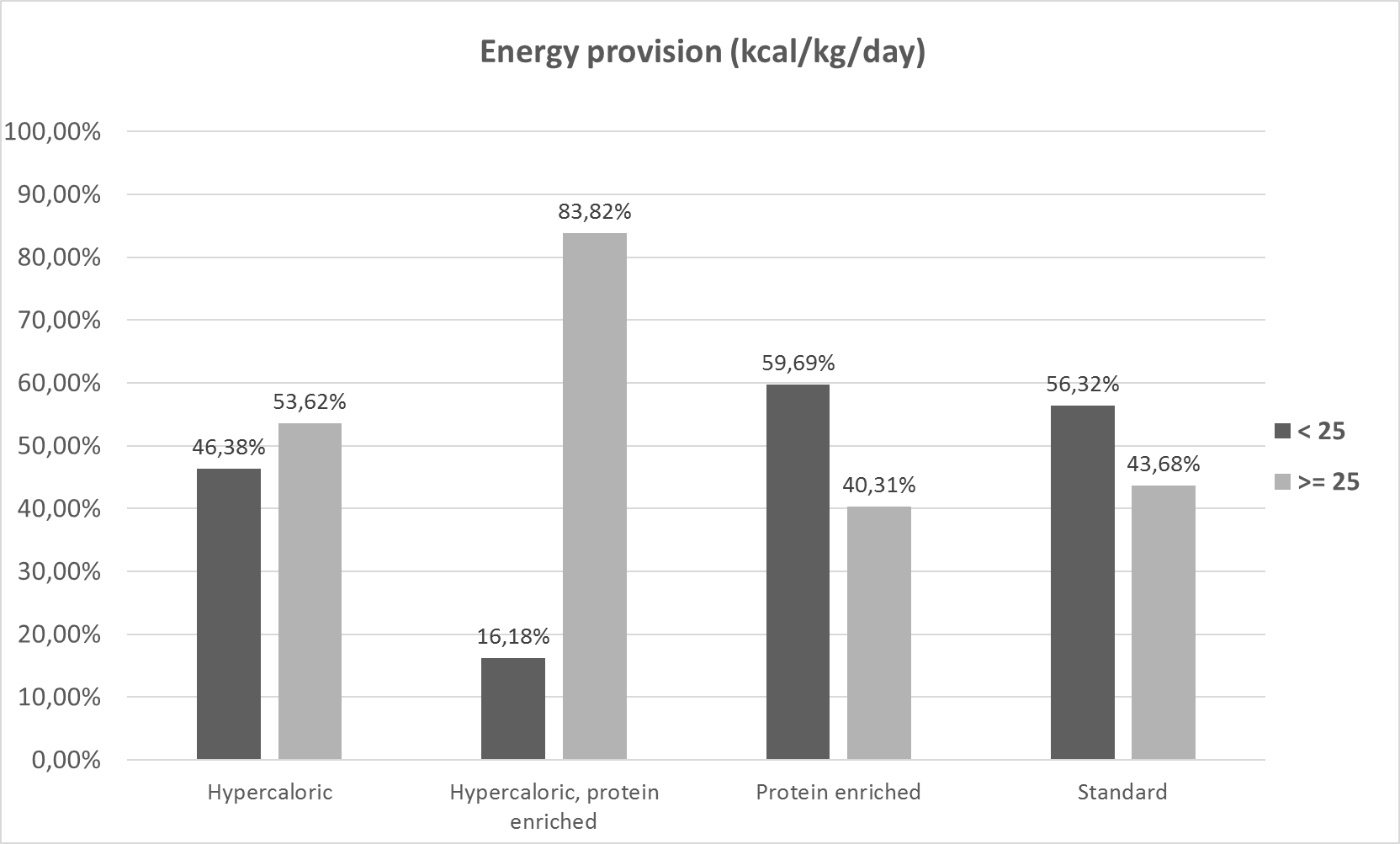


Figure 3. Energy provision and FSMP type in general population


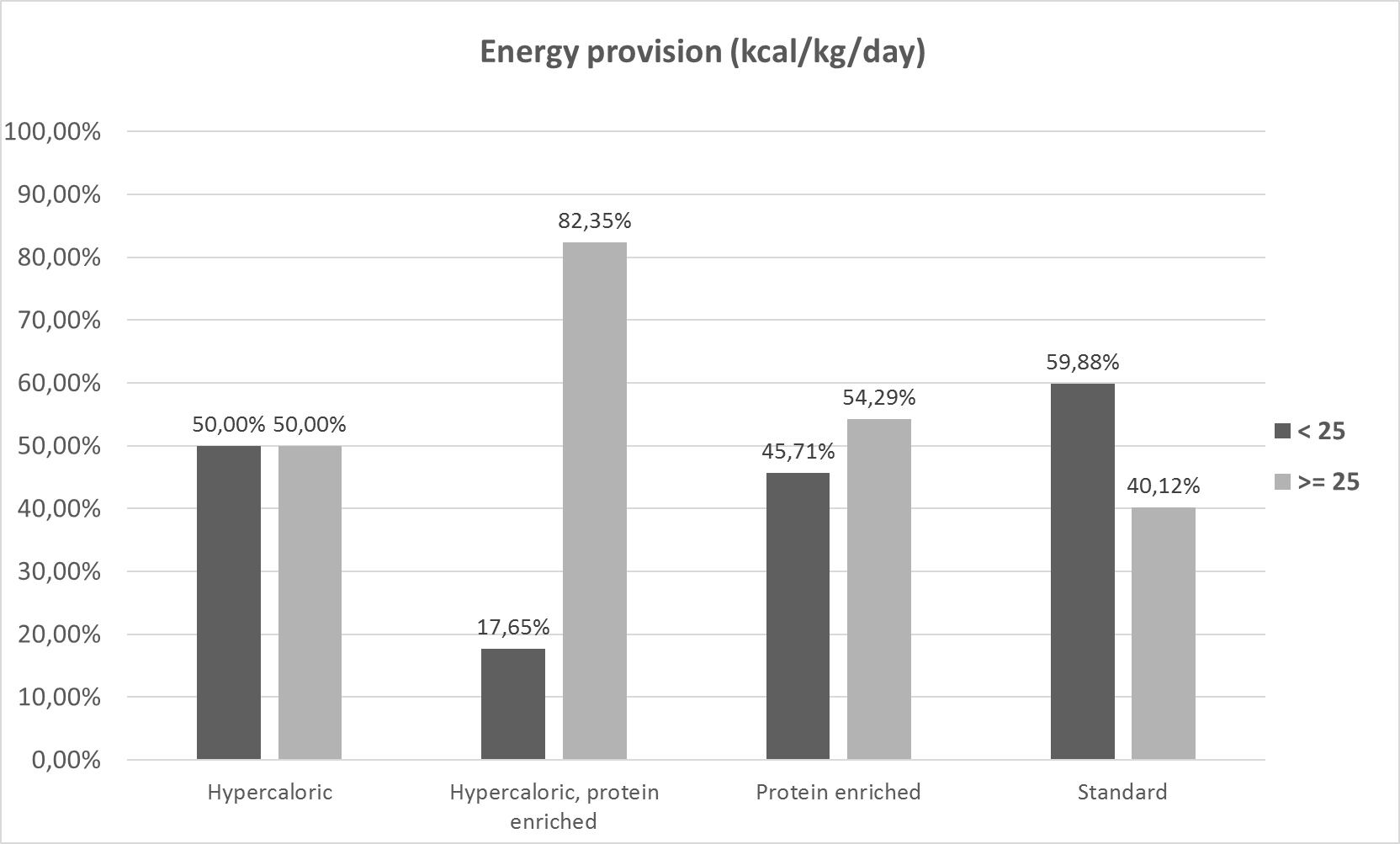


Figure 4. Energy provision and FSMP type in cancer patients

Table 2. Micronutrient provision (left column- according to the main indication for HEN, right column- according to the FSMP type)

| **Main indication for HEN** | | | | |  | **FSMP type** | | | | |
| --- | --- | --- | --- | --- | --- | --- | --- | --- | --- | --- |
| **General population (n=772)** | | | | |  | **Standard (n=505)** | | | | |
|  | med. | q1 | q3 | sd |  |  | med. | q1 | q3 | sd |
| Vitamin A: | 141,43% | 117,14% | 175,71% | 46,93% |  | Vitamin A: | 136,67% | 117,14% | 170,00% | 51,16% |
| Vitamin D: | 98,67% | 66,67% | 121,33% | 36% |  | Vitamin D: | 100,00% | 66,67% | 130,00% | 37,42% |
| Vitamin E: | 200,25% | 162,5% | 300% | 262,24% |  | Vitamin E: | 225,00% | 187,50% | 320,00% | 363,99% |
| Vitamin K: | 122,31% | 103,08% | 154,62% | 43,95% |  | Vitamin K: | 122,31% | 98,46% | 153,46% | 52,39% |
| Vitamin B1 (Thiamin): | 152% | 127,27% | 181,82% | 46,72% |  | Vitamin B1 (Thiamin): | 161,54% | 136,36% | 200,00% | 52,36% |
| Vitamin B2 (Riboflavin): | 181,82% | 146,15% | 218,18% | 55,23% |  | Vitamin B2 (Riboflavin): | 184,62% | 154,55% | 230,77% | 274,05% |
| Vitamin B3 (Niacin): | 100% | 66,56% | 128,57% | 47,97% |  | Vitamin B3 (Niacin): | 96,43% | 63,39% | 137,14% | 54,15% |
| Vitamin B6: | 141,18% | 117,65% | 170% | 45,81% |  | Vitamin B6: | 144,00% | 117,65% | 176,47% | 63,37% |
| Vitamin B9 (Folate): | 87,5% | 72,5% | 101,25% | 26,12% |  | Vitamin B9 (Folate): | 84,38% | 70,00% | 101,25% | 29,46% |
| Vitamin B12: | 140% | 112,5% | 168,75% | 53,88% |  | Vitamin B12: | 140,63% | 112,50% | 196,88% | 74,12% |
| Vitamin B7 (Biotin): | 183,33% | 153,33% | 233,33% | 66,63% |  | Vitamin B7 (Biotin): | 186,67% | 153,33% | 250,00% | 69,83% |
| Vitamin C: | 153,85% | 119,11% | 200% | 142,07% |  | Vitamin C: | 160,00% | 122,22% | 200,00% | 144,65% |
| **Neurological diseases (n=425)** | | | | |  | **Hypercaloric (n=69)** | | | | |
| Vitamin A: | 146,43% | 117,14% | 175,71% | 47,17% |  | Vitamin A: | 136,17% | 109,33% | 170,00% | 52,87% |
| Vitamin D: | 100% | 66,67% | 122,5% | 35,84% |  | Vitamin D: | 100,00% | 70,00% | 130,00% | 37,52% |
| Vitamin E: | 203,13% | 162,5% | 300% | 271,67% |  | Vitamin E: | 237,50% | 182,00% | 333,75% | 379,36% |
| Vitamin K: | 122,31% | 103,08% | 154,62% | 44,87% |  | Vitamin K: | 120,00% | 96,36% | 145,45% | 54,45% |
| Vitamin B1 (Thiamin): | 150% | 127,27% | 181,82% | 49,03% |  | Vitamin B1 (Thiamin): | 163,64% | 136,36% | 195,15% | 53,35% |
| Vitamin B2 (Riboflavin): | 182,69% | 145,45% | 218,18% | 57,27% |  | Vitamin B2 (Riboflavin): | 181,82% | 145,45% | 231,82% | 346,52% |
| Vitamin B3 (Niacin): | 98,75% | 65% | 128,57% | 49,3% |  | Vitamin B3 (Niacin): | 86,67% | 55,63% | 128,57% | 55,17% |
| Vitamin B6: | 90,63% | 72,5% | 101,25% | 27,26% |  | Vitamin B6: | 141,67% | 117,65% | 196,92% | 78,17% |
| Vitamin B9 (Folate): | 150% | 112,5% | 180% | 54,87% |  | Vitamin B9 (Folate): | 75,00% | 70,00% | 100,13% | 26,93% |
| Vitamin B12: | 182% | 153,33% | 245% | 68,78% |  | Vitamin B12: | 140,63% | 109,38% | 187,50% | 79,79% |
| Vitamin B7 (Biotin): | 155,56% | 122,22% | 200% | 146,94% |  | Vitamin B7 (Biotin): | 166,67% | 153,33% | 250,00% | 69,26% |
| Vitamin C: | 141,18% | 116,47% | 170% | 47,56% |  | Vitamin C: | 160,00% | 122,22% | 200,00% | 110,07% |
| **Cancer (n=259)** | | | | |  | **Protein enriched (n=129)** | | | | |
| Vitamin A: | 142,22% | 117,14% | 175,71% | 47,98% |  | Vitamin A: | 123,40% | 102,22% | 164,29% | 42,51% |
| Vitamin D: | 93,33% | 66,67% | 120% | 35,62% |  | Vitamin D: | 86,67% | 66,67% | 100,00% | 31,07% |
| Vitamin E: | 200% | 162,5% | 260% | 231,31% |  | Vitamin E: | 200,00% | 160,00% | 267,00% | 321,08% |
| Vitamin K: | 122,31% | 103,08% | 154,62% | 43,5% |  | Vitamin K: | 114,15% | 96,36% | 140,00% | 39,36% |
| Vitamin B1 (Thiamin): | 153,85% | 127,27% | 181,82% | 44,68% |  | Vitamin B1 (Thiamin): | 136,36% | 115,38% | 173,08% | 44,85% |
| Vitamin B2 (Riboflavin): | 181,82% | 147,69% | 218,18% | 53,81% |  | Vitamin B2 (Riboflavin): | 154,55% | 136,36% | 200,00% | 183,72% |
| Vitamin B3 (Niacin): | 100% | 70,71% | 128,57% | 47,04% |  | Vitamin B3 (Niacin): | 97,50% | 62,13% | 115,00% | 42,87% |
| Vitamin B6: | 83,44% | 70% | 101,25% | 25,03% |  | Vitamin B6: | 130,77% | 113,33% | 160,00% | 42,67% |
| Vitamin B9 (Folate): | 131,25% | 106,67% | 168,75% | 53,67% |  | Vitamin B9 (Folate): | 75,00% | 67,50% | 100,00% | 22,76% |
| Vitamin B12: | 180% | 153,33% | 232% | 63,97% |  | Vitamin B12: | 131,25% | 104,17% | 166,67% | 58,85% |
| Vitamin B7 (Biotin): | 146,67% | 111,67% | 184,72% | 124,67% |  | Vitamin B7 (Biotin): | 166,67% | 133,33% | 200,00% | 54,58% |
| Vitamin C: | 141,18% | 120% | 170% | 43,16% |  | Vitamin C: | 134,00% | 111,11% | 186,67% | 150,88% |
| **Other (n=88)** | | | | |  | **Hypercaloric, protein enriched (n=68)** | | | | |
| Vitamin A: | 136,67% | 117,14% | 172,86% | 42,72% |  | Vitamin A: | 122,22% | 102,22% | 155,24% | 46,30% |
| Vitamin D: | 93,33% | 66,67% | 125% | 38,21% |  | Vitamin D: | 86,67% | 66,67% | 106,17% | 29,44% |
| Vitamin E: | 205,56% | 176,17% | 300% | 295,49% |  | Vitamin E: | 195,00% | 148,13% | 240,00% | 258,43% |
| Vitamin K: | 121,82% | 97,41% | 153,85% | 40,93% |  | Vitamin K: | 108,39% | 96,36% | 134,62% | 39,77% |
| Vitamin B1 (Thiamin): | 150% | 122,73% | 176,92% | 40,9% |  | Vitamin B1 (Thiamin): | 145,83% | 115,38% | 177,73% | 47,15% |
| Vitamin B2 (Riboflavin): | 181,82% | 145,8% | 205,45% | 49,37% |  | Vitamin B2 (Riboflavin): | 158,46% | 130,77% | 196,15% | 132,58% |
| Vitamin B3 (Niacin): | 96,43% | 64,29% | 125% | 44,26% |  | Vitamin B3 (Niacin): | 100,00% | 66,25% | 120,00% | 45,68% |
| Vitamin B6: | 87,75% | 72,5% | 101,25% | 23,27% |  | Vitamin B6: | 144,31% | 125,00% | 184,62% | 48,91% |
| Vitamin B9 (Folate): | 131,25% | 104,17% | 174,38% | 48,72% |  | Vitamin B9 (Folate): | 77,55% | 67,50% | 100,06% | 24,36% |
| Vitamin B12: | 186,67% | 149,31% | 230% | 64,24% |  | Vitamin B12: | 130,73% | 107,19% | 167,71% | 53,84% |
| Vitamin B7 (Biotin): | 166,67% | 122,22% | 200% | 162,51% |  | Vitamin B7 (Biotin): | 166,67% | 153,33% | 200,00% | 52,52% |
| Vitamin C: | 140% | 115,49% | 184,62% | 45,34% |  | Vitamin C: | 133,33% | 111,11% | 183,33% | 133,24% |

| **General population (n=772)** | | | | |  | **Standard (n=505)** | | | | |
| --- | --- | --- | --- | --- | --- | --- | --- | --- | --- | --- |
|  | med. | q1 | q3 | sd |  |  | med. | q1 | q3 | sd |
| Sodium: | 66,67% | 53,33% | 86,67% | 25,11% |  | Sodium: | 66,67% | 53,33% | 90,00% | 26,11% |
| Potassium: | 48,57% | 40,86% | 60,00% | 14,83% |  | Potassium: | 49,77% | 42,86% | 63,71% | 15,44% |
| Chloride: | 54,35% | 41,30% | 67,93% | 18,41% |  | Chloride: | 54,35% | 43,48% | 70,65% | 18,84% |
| Calcium: | 81,67% | 66,67% | 104,00% | 36,84% |  | Calcium: | 83,33% | 66,67% | 106,25% | 39,42% |
| Phosphorus: | 108,00% | 90,00% | 139,43% | 35,49% |  | Phosphorus: | 112,50% | 92,86% | 150,00% | 36,84% |
| Magnesium: | 71,43% | 54,76% | 86,25% | 22,67% |  | Magnesium: | 71,88% | 57,14% | 89,29% | 22,79% |
| Iron: | 160,00% | 130,00% | 205,40% | 63,20% |  | Iron: | 165,00% | 130,00% | 224,00% | 64,53% |
| Zinc: | 156,25% | 125,00% | 200,00% | 62,21% |  | Zinc: | 163,64% | 130,28% | 204,55% | 62,58% |
| Copper: | 216,67% | 173,33% | 280,00% | 73,58% |  | Copper: | 222,22% | 180,56% | 283,33% | 76,05% |
| Manganese: | 173,91% | 130,43% | 215,22% | 64,73% |  | Manganese: | 180,00% | 133,33% | 220,00% | 65,14% |
| Fluoride: | 39,00% | 32,50% | 50,00% | 15,87% |  | Fluoride: | 40,63% | 33,33% | 50,00% | 16,22% |
| Molybdenum: | 288,89% | 222,22% | 333,33% | 96,85% |  | Molybdenum: | 288,89% | 222,22% | 333,33% | 99,95% |
| Selenium: | 155,45% | 121,82% | 182,73% | 53,48% |  | Selenium: | 155,45% | 126,55% | 190,91% | 55,01% |
| Chromium: | 335,00% | 250,00% | 428,57% | 145,09% |  | Chromium: | 335,00% | 268,00% | 428,57% | 140,35% |
| Iodine: | 107,57% | 86,67% | 133,00% | 35,13% |  | Iodine: | 110,83% | 88,67% | 133,00% | 36,93% |
| **Neurology (n=425)** | | | | |  | **Hypercaloric (n=69)** | | | | |
| Sodium: | 66,67% | 53,33% | 86,67% | 24,94% |  | Sodium: | 66,67% | 53,33% | 83,33% | 26,39% |
| Potassium: | 48,93% | 40,96% | 60,00% | 14,43% |  | Potassium: | 48,57% | 40,86% | 54,86% | 14,43% |
| Chloride: | 54,35% | 40,76% | 67,93% | 18,14% |  | Chloride: | 54,35% | 39,42% | 65,22% | 16,36% |
| Calcium: | 82,54% | 66,67% | 104,00% | 39,90% |  | Calcium: | 80,00% | 66,67% | 100,00% | 36,87% |
| Phosphorus: | 108,00% | 90,00% | 144,00% | 38,23% |  | Phosphorus: | 104,29% | 90,00% | 139,29% | 33,45% |
| Magnesium: | 71,43% | 54,80% | 86,25% | 23,25% |  | Magnesium: | 71,88% | 59,52% | 89,84% | 23,70% |
| Iron: | 162,65% | 130,00% | 208,00% | 66,42% |  | Iron: | 165,00% | 130,00% | 200,00% | 57,45% |
| Zinc: | 163,64% | 125,00% | 217,84% | 65,14% |  | Zinc: | 163,64% | 125,00% | 204,55% | 56,88% |
| Copper: | 220,00% | 183,33% | 283,33% | 77,41% |  | Copper: | 216,67% | 180,56% | 277,78% | 66,56% |
| Manganese: | 176,09% | 130,80% | 217,39% | 67,44% |  | Manganese: | 183,33% | 133,33% | 225,00% | 64,84% |
| Fluoride: | 39,00% | 32,50% | 50,00% | 15,91% |  | Fluoride: | 40,00% | 32,50% | 53,33% | 17,90% |
| Molybdenum: | 288,89% | 222,22% | 333,33% | 99,83% |  | Molybdenum: | 288,89% | 244,44% | 333,33% | 87,59% |
| Selenium: | 155,45% | 124,36% | 182,73% | 55,09% |  | Selenium: | 155,45% | 127,27% | 182,73% | 47,72% |
| Chromium: | 335,00% | 266,67% | 440,00% | 146,62% |  | Chromium: | 335,00% | 250,00% | 500,00% | 153,54% |
| Iodine: | 108,33% | 86,67% | 133,00% | 36,93% |  | Iodine: | 108,33% | 86,67% | 133,33% | 34,04% |
| **Cancer (n=259)** | | | | |  | **Protein enriched (n=129)** | | | | |
| Sodium: | 66,67% | 53,33% | 87,33% | 25,66% |  | Sodium: | 66,67% | 53,33% | 80,00% | 21,43% |
| Potassium: | 48,57% | 39,43% | 60,00% | 15,46% |  | Potassium: | 42,86% | 38,57% | 53,57% | 12,37% |
| Chloride: | 54,35% | 42,61% | 67,93% | 18,96% |  | Chloride: | 53,33% | 39,13% | 64,00% | 18,09% |
| Calcium: | 82,00% | 66,67% | 102,50% | 32,52% |  | Calcium: | 70,83% | 66,67% | 93,33% | 27,54% |
| Phosphorus: | 107,14% | 90,00% | 135,00% | 31,79% |  | Phosphorus: | 102,86% | 85,71% | 128,57% | 30,98% |
| Magnesium: | 71,43% | 54,76% | 89,29% | 22,11% |  | Magnesium: | 62,50% | 53,13% | 76,67% | 21,76% |
| Iron: | 160,00% | 120,00% | 200,00% | 59,45% |  | Iron: | 156,00% | 110,00% | 190,00% | 54,44% |
| Zinc: | 152,73% | 125,00% | 187,50% | 58,26% |  | Zinc: | 136,36% | 109,09% | 181,82% | 61,50% |
| Copper: | 216,67% | 166,67% | 277,78% | 69,41% |  | Copper: | 200,00% | 150,00% | 236,11% | 64,50% |
| Manganese: | 166,67% | 127,78% | 215,22% | 62,31% |  | Manganese: | 150,00% | 117,39% | 187,83% | 58,30% |
| Fluoride: | 39,00% | 31,25% | 50,00% | 16,29% |  | Fluoride: | 33,33% | 30,00% | 48,75% | 12,83% |
| Molybdenum: | 277,78% | 222,22% | 333,33% | 93,82% |  | Molybdenum: | 263,33% | 222,22% | 333,33% | 83,44% |
| Selenium: | 146,18% | 121,82% | 182,73% | 51,13% |  | Selenium: | 132,05% | 114,55% | 174,55% | 47,69% |
| Chromium: | 335,00% | 229,71% | 418,75% | 141,83% |  | Chromium: | 335,00% | 223,33% | 443,33% | 159,54% |
| Iodine: | 106,40% | 86,67% | 130,00% | 32,96% |  | Iodine: | 92,89% | 86,67% | 130,00% | 27,63% |
| **Other (n=88)** | | | | |  | **Hypercaloric, protein enriched (n=68)** | | | | |
| Sodium: | 66,67% | 55,33% | 83,38% | 24,57% |  | Sodium: | 66,67% | 53,33% | 80,00% | 20,56% |
| Potassium: | 47,25% | 42,86% | 57,86% | 15,00% |  | Potassium: | 43,57% | 38,57% | 57,29% | 13,66% |
| Chloride: | 54,35% | 40,76% | 66,30% | 18,26% |  | Chloride: | 50,00% | 42,78% | 61,25% | 16,56% |
| Calcium: | 80,00% | 66,67% | 105,00% | 33,34% |  | Calcium: | 82,67% | 80,00% | 102,50% | 28,82% |
| Phosphorus: | 104,29% | 90,00% | 139,71% | 31,51% |  | Phosphorus: | 104,57% | 90,00% | 133,71% | 32,02% |
| Magnesium: | 71,65% | 54,46% | 82,53% | 21,62% |  | Magnesium: | 63,89% | 54,84% | 81,55% | 21,05% |
| Iron: | 160,00% | 125,00% | 200,00% | 56,49% |  | Iron: | 130,00% | 110,00% | 182,71% | 59,06% |
| Zinc: | 151,36% | 125,00% | 204,55% | 59,14% |  | Zinc: | 132,12% | 109,09% | 163,64% | 59,04% |
| Copper: | 222,22% | 173,81% | 280,00% | 65,06% |  | Copper: | 200,00% | 159,72% | 248,33% | 70,12% |
| Manganese: | 173,91% | 132,01% | 215,22% | 57,84% |  | Manganese: | 143,48% | 117,39% | 191,58% | 65,34% |
| Fluoride: | 37,50% | 32,50% | 50,00% | 14,52% |  | Fluoride: | 33,57% | 30,63% | 45,97% | 14,23% |
| Molybdenum: | 288,89% | 222,22% | 333,33% | 90,39% |  | Molybdenum: | 266,67% | 222,22% | 333,33% | 94,28% |
| Selenium: | 154,09% | 121,82% | 182,73% | 51,78% |  | Selenium: | 127,27% | 103,64% | 159,09% | 50,85% |
| Chromium: | 334,17% | 268,00% | 418,75% | 147,24% |  | Chromium: | 273,58% | 191,43% | 392,86% | 128,77% |
| Iodine: | 104,00% | 86,67% | 130,00% | 32,24% |  | Iodine: | 97,22% | 86,67% | 130,00% | 29,77% |

Table 3. Rate of patients administered with more than 100% RDA

|  | **Vitamin A** | **Vitamin D** | **Vitamin E** | **Vitamin K** | **Vitamin B1 (Thiamin)** | **Vitamin B2 (Riboflavin)** | **Vitamin B3 (Niacin)** | **Vitamin B5** | **Vitamin B6** | **Vitamin B9 (Folate)** | **Vitamin B12** | **Vitamin B7 (Biotin)** | **Vitamin C** |  |  |
| --- | --- | --- | --- | --- | --- | --- | --- | --- | --- | --- | --- | --- | --- | --- | --- |
| Other | 86,36% | 43,18% | 97,73% | 73,86% | 96,59% | 97,73% | 50,00% | 38,64% | 95,45% | 32,95% | 88,64% | 100,00% | 89,77% |  |  |
| Neurology | 90,57% | 52,12% | 99,29% | 78,77% | 97,64% | 98,82% | 50,00% | 37,74% | 92,45% | 42,22% | 90,33% | 99,53% | 88,44% |  |  |
| Cancer | 86,87% | 47,10% | 99,23% | 78,76% | 97,30% | 99,23% | 52,90% | 31,27% | 92,66% | 38,61% | 88,80% | 99,61% | 88,42% |  |  |
| All | 88,85% | 49,42% | 99,09% | 78,21% | 97,41% | 98,83% | 50,97% | 35,67% | 92,87% | 39,95% | 89,62% | 99,61% | 88,59% |  |  |
|  |  |  |  |  |  |  |  |  |  |  |  |  |  |  |  |
|  | **Sodium** | **Potassium** | **Chloride** | **Calcium** | **Phosphorus** | **Magnesium** | **Iron** | **Zinc** | **Copper** | **Manganese** | **Fluoride** | **Molybdenum** | **Selenium** | **Chromium** | **Iodine** |
| Other | 17,05% | 1,14% | 1,14% | 29,55% | 71,59% | 11,36% | 95,45% | 90,91% | 100,00% | 94,32% | 0,00% | 95,45% | 89,77% | 97,73% | 51,14% |
| Neurology | 19,58% | 0,24% | 1,65% | 33,02% | 72,41% | 12,74% | 95,28% | 91,27% | 98,82% | 93,16% | 1,18% | 96,46% | 91,27% | 98,58% | 58,25% |
| Cancer | 20,85% | 1,16% | 1,93% | 33,98% | 67,95% | 13,13% | 93,44% | 92,28% | 98,46% | 91,51% | 0,77% | 95,37% | 89,96% | 97,68% | 56,37% |
| All | 19,71% | 0,65% | 1,69% | 32,94% | 70,82% | 12,71% | 94,68% | 91,57% | 98,83% | 92,74% | 0,91% | 95,98% | 90,66% | 98,18% | 56,81% |

Table 4. Reference values for micronutrient (based on the recommendations of Polish National Institute of Public Health - National Institute of Hygiene and European Food Safety Authority -EFSA (16,19)

| **Micronutrient** | **Male** | **Female** |
| --- | --- | --- |
| Vitamin A (µg/d) | 900 | 700 |
| Vitamin D (µg/d) | 15 | 15 |
| Vitamin E (mg/d) | 10 | 8 |
| Vitamin K (µg/d) | 65 | 55 |
| Vitamin B1 (Thiamin) (mg/d) | 1,3 | 1,1 |
| Vitamin B2 (Riboflavin) (mg/d) | 1,3 | 1,1 |
| Vitamin B3 (Niacin) (mg/d) | 16 | 14 |
| Vitamin B6 (mg/d) | 1,7 | 1,5 |
| Vitamin B9 (Folate) (µg/d) | 400 | 400 |
| Vitamin B12 (µg/d) | 2,4 | 2,4 |
| Vitamin B7 (Biotin) (µg/d) | 30 | 30 |
| Vitamin C (mg/d) | 90 | 75 |
| Sodium (mg/d) | 1500 | 1500 |
| Potassium (mg/d) | 3500 | 3500 |
| Chloride (mg/d) | 2300 | 2300 |
| Calcium (mg/d) | 1200 | 1200 |
| Phosphorus (mg/d) | 700 | 700 |
| Magnesium (mg/d) | 420 | 320 |
| Iron (mg/d) | 10 | 10 |
| Zinc (mg/d) | 11 | 8 |
| Copper (mg/d) | 0,9 | 0,9 |
| Manganese (mg/d) | 2,3 | 1,8 |
| Fluoride (mg/d) | 4 | 3 |
| Molybdenum (µg/d) | 45 | 45 |
| Selenium (µg/d) | 55 | 55 |
| Chromium (µg/d) | 30 | 20 |
| Iodine (µg/d) | 150 | 150 |
